# Multi-Input Logic-in-Memory for Ultra-Low Power Non-Von Neumann Computing

**DOI:** 10.3390/mi12101243

**Published:** 2021-10-14

**Authors:** Tommaso Zanotti, Paolo Pavan, Francesco Maria Puglisi

**Affiliations:** Department of Engineering “Enzo Ferrari”, University of Modena and Reggio Emilia, Via P. Vivarelli 10/1, 41125 Modena, Italy; paolo.pavan@unimore.it (P.P.); francescomaria.puglisi@unimore.it (F.M.P.)

**Keywords:** implication logic, logic-in-memory, memristor, Boolean algebra, RRAM, BNN

## Abstract

Logic-in-memory (LIM) circuits based on the material implication logic (IMPLY) and resistive random access memory (RRAM) technologies are a candidate solution for the development of ultra-low power non-von Neumann computing architectures. Such architectures could enable the energy-efficient implementation of hardware accelerators for novel edge computing paradigms such as binarized neural networks (BNNs) which rely on the execution of logic operations. In this work, we present the multi-input IMPLY operation implemented on a recently developed smart IMPLY architecture, SIMPLY, which improves the circuit reliability, reduces energy consumption, and breaks the strict design trade-offs of conventional architectures. We show that the generalization of the typical logic schemes used in LIM circuits to multi-input operations strongly reduces the execution time of complex functions needed for BNNs inference tasks (e.g., the 1-bit Full Addition, XNOR, Popcount). The performance of four different RRAM technologies is compared using circuit simulations leveraging a physics-based RRAM compact model. The proposed solution approaches the performance of its CMOS equivalent while bypassing the von Neumann bottleneck, which gives a huge improvement in bit error rate (by a factor of at least 10^8^) and energy-delay product (projected up to a factor of 10^10^).

## 1. Introduction

With the number of connected devices in use exceeding 17 billion, the volume of exchanged data rapidly rises. From this standpoint, edge computing ensures a decrease in the amount of data to be exchanged, relaxing data transfer and power constraints with obvious benefits for consumer and industrial Internet of Things (IoT), smart cities, artificial intelligence (AI), machine learning, and 5G industry. Still, its implementation requires ultra-low power hardware solutions, mainly hindered by the von Neumann bottleneck (VNB) [1,2,3], i.e., the slow and energy-hungry data transfer between CPUs and off-chip non-volatile memories. As suggested in the latest IRDS report [4], logic-in-memory (LIM) architectures that allow executing Boolean operations directly inside the memory could circumvent the VNB. Developing LIM hardware accelerators would enable the deployment at the edge of powerful and data-intensive computing paradigms such as binarized neural networks (BNNs) [5,6,7] and hyperdimensional computing [8,9,10], which strongly rely on the energy-efficient execution of logic operations. Among LIM solutions [11,12,13,14,15,16,17], circuits based on resistive memory (RRAM) technology and the implication logic (IMPLY) offer ultra-dense back end of line (BEOL) integration. Currently, the main showstoppers [12,18] hindering the introduction of RRAM-based LIM circuits are the high energy per operation (as compared to CMOS gates), the degradation of the logic values of RRAMs during circuit operation, and the need to apply very precise voltage pulses (mV accuracy may be required [12,18,19]). Moreover, while in CMOS logic multiple operations can be computed in parallel on the same inputs, in IMPLY-based LIM circuits operations are carried out sequentially. Thus, reducing the execution time is critical. Recently a novel low-power non-von Neumann LIM solution, called smart-IMPLY (SIMPLY) [20], was introduced and shown to reduce the energy per operation, to solve the problem of logic state degradation of RRAM cells, and to eliminate the need for very precise voltage pulses. Moreover, active research interest has been directed towards the study of circuit implementations of the multi-input IMPLY operation, as Siemon et al. [21] proposed the three-input IMPLY operation (named ORNOR by the authors). However, studies on circuit implementations considering the IMPLY operation generalized to more than three inputs have never been presented. 

**Figure 1:**
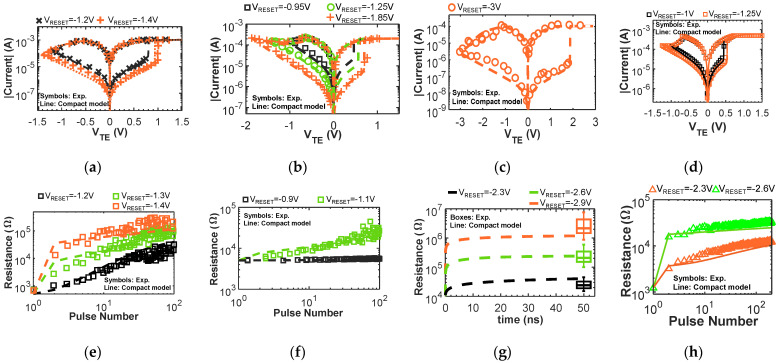
(**a**,**e**) Simulated (lines) and experimental (symbols) results of a multi-layer Pt/TiO_x_/HfO_x_/TiO_x_/HfO_x_/TiN RRAM formed at I_C_ = 1 mA, technology 1 in this work. (**a**) DC I-V curves at different reset voltages. (**e**) Pulsed reset curves using a train of 10 ns pulses with different voltages. Data from [25]. (**b**,**f**) Simulated (lines) and experimental (symbols) results of a mono-layer TiN/HfO_2_/Ti/TiN RRAM formed at I_C_ = 200 µA, technology 2 in this work. (**b**) DC I-V curves at different reset voltages. (**f**) Pulsed reset curves using a train of 1 µs pulses with different voltages. Data from [27]. (**c**,**g**) Simulated (lines) and experimental (symbols) results of a bi-layer TiN/HfO_x_/AlO_x_/Pt RRAM formed at I_C_ = 100 µA, technology 3 in this work. (**c**) DC I-V curve. (**g**) Pulsed reset curves using a single 50 ns pulse with different voltages. Data from [29]. (**d**,**h**) Simulated (lines) and experimental (symbols) results of a TiN/Ti/HfO_x_/TiN from SEMATECH [22] RRAM formed at I_C_ = 500 µA, technology 4 in this work. (**d**) DC I-V curve. (**h**) Pulsed reset curves using a train of 10 µs pulses with different voltages. Data collected experimentally.

In this paper, we demonstrate the multi-input IMPLY logic scheme in the framework of SIMPLY architecture by exploiting multi-input (>2) operations, strongly reducing the execution time of complex logic functions (e.g., the set of logic operations to implement BNN inference tasks). We experimentally verify the correct circuit functionality of the core operation used in SIMPLY on TiN/Ti/HfO_x_/TiN devices from SEMATECH [22] (technology 4 in this work, electrical characteristics shown in Figure 1d,h). Then, for accurate and trustable results circuit simulations are performed using a fully physics-based RRAM compact model from [23,24] that includes thermal effects (also self-heating), variability, and multilevel random telegraph noise (RTN). We demonstrate the feasibility of the proposed architecture by comparing the performance obtained with three RRAM technologies from the literature (a multi-layer Pt/TiO_x_/HfO_x_/TiO_x_/HfO_x_/TiN RRAM referred to in this work as technology 1 [25,26], a TiN/HfO_2_/Ti/TiN RRAM technology 2 [27,28], and a TiN/HfO_x_/AlO_x_/Pt RRAM technology 3 [29]). The results obtained with all the technologies show how combining the proposed innovations allows realizing LIM circuits that outperform both existing LIM solutions and CMOS gates performance when the VNB time and energy overhead is considered, coming close to CMOS gates performance alone (excluding the VNB). Finally, we benchmark the performance improvement brought by the introduction of the multi-input IMPLY operation when executing a BNN inference task with respect to the previous IMPLY-based implementation from [30]. 

## 2. Materials and Methods

### 2.1. RRAM Physics-Based Compact Model for Circuit Simulations

**Figure 2:**
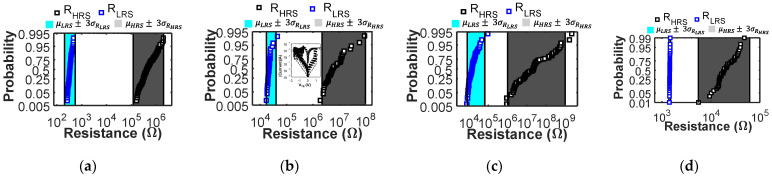
Probability plots showing the experimental (cyan and grey bands) and simulated (blue and black squares) cycle to cycle variability. Cyan and grey areas indicate the range µ±3σ of the experimental LRS and HRS distributions. For each technology, the simulations replicate the available experimental conditions, and the reset and set voltages are reported. Specifically, (**a**) reports the variability obtained with technology 1 under quasi-static DC conditions (simulation variability parameters σ_x_ = 0.35 nm, σ_S_ = 2.7 nm^2^). Data from [26]; (**b**) reports the variability obtained with technology 2 under quasi-static DC conditions and I_C_ = 20 µA, as for this technology experimental variability data [28] are available only at I_C_ = 20 µA (variability parameters σ_x_ = 0.8 nm, σ_S_ = 24.9 nm^2^). The inset shows the experimental (square symbols) and simulated (lines) DC IV curves, where only *k_cf_* (*k_cf_*_ 20µA_ = *k_cf_*_ 200µA_/100), *k_ex_* (*k_ex_*_ 20µA_ = *k_ex_*_ 200µA_/10), and *S* (*S*_20__µA_ = *S*_200__µA_/10) parameters were changed to account for the different characteristic of the very narrow CF obtained with such low current compliance; (**c**) reports the variability obtained with technology 3 [29] under quasi-static DC conditions (variability parameters σ_x_ = 0.81 nm, σ_S_ = 0.7785 nm^2^); (**d**) reports the variability experimentally measured on technology 4. HRS variability data are obtained using 10 µs reset voltage pulses, while LRS data with a quasi-static positive voltage ramp as this was the only way to provide an accurate current compliance with our test setup. (Variability parameters σ_x_ = 0.7 nm, σ_S_ = 0.350 nm^2^).

To study LIM circuits, physics-based compact models that include device non-idealities, such as device-to-device and cycle-to-cycle variability, RTN, self-heating, and thermal effects, represent a very important tool that enables achieving accurate results [18] through circuit simulations. Neglecting RRAMs nonidealities can easily result in poor circuit designs [12,18,31] with low reliability or circuits that do not work at all. In this work, we use the Verilog-A RRAM compact model from [23], which is fully physics-based and supported by the results of advanced physical multi-scale simulations. The total device resistance is modeled as the sum of a conductive filament (CF) and a dielectric barrier contribution. The barrier thickness is modeled dynamically with differential equations that reproduce the device behavior during the reset operation by considering the field-driven oxygen ions drift and recombination, and during the set operation by considering the field-accelerated bond breaking and related defect generation [23,24]. Moreover, thermal effects are modeled dynamically, considering both the thermal conductance and thermal capacitance of the CF and the barrier, thus enabling accurate predictions also when using very short pulses [18]. The model includes the intrinsic variability of both resistive states and the effects of multilevel RTN and its statistical variations [24]. In particular, to introduce cycle-to-cycle variability we add appropriate zero-mean normally distributed random noise sources on the dielectric barrier thickness during reset, and on the CF cross-section while performing a set transition [23,24]. The model, for all the four different RRAM technologies [22,25,27,29] (see Figure 1a–h) explored in this work, correctly reproduces the quasi-static IV, the response to fast reset pulses, and the experimental cycle-to-cycle variability using a single set of parameters per technology (see Figure 1 and Figure 2), thus highlighting the quality of the modeling approach and avoiding the need to design multiple parameter calibrations to reproduce the device behavior under different operating conditions. Technology 4 was experimentally characterized using the Keithley 4200-SCS parameter analyzer.

The calibrated compact model was used to perform circuit simulations with the Cadence Virtuoso^®^ software, to determine the performance and reliability of different LIM solutions. 

### 2.2. Logic-in-Memory with RRAM Devices and the Material Implication Logic

**Figure 3:**
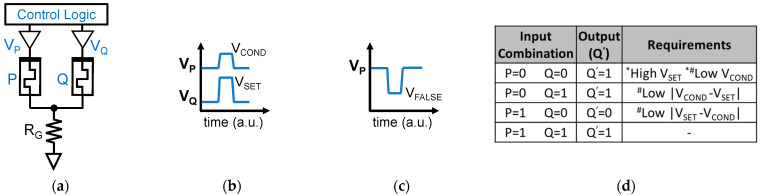
(**a**) Schematic of the IMPLY gate. (**b**) Pulses applied to the top electrodes of P (V_P_ = V_COND_) and Q (V_Q_ = V_SET_) to execute the IMPLY operation. (**c**) Pulse applied at the top electrode of P (V_P_ = V_FALSE_) to perform the FALSE P operation. (**d**) Truth table of the IMPLY operation where the conflicting V_SET_ and V_COND_ requirements for gate functionality (*) and reduced degradation (#) for the four input combinations are evidenced.

The material implication logic is a functionally complete logic that can be effectively implemented with RRAM devices [11]. In fact, RRAMs enable its efficient implementation using a circuit architecture like the one in Figure 3a. A control logic equipped with analog tri-state buffers is needed to deliver appropriate voltages at the top electrode (TE) of each RRAM device of the array, with the devices in the array having their bottom electrodes (BE) connected to the same resistor R_G_. Differently from traditional logic gates, in RRAM-based LIM schemes, the logic values are not encoded as voltages at circuit nodes, but as nonvolatile resistive states of RRAMs (HRS for logic 0, LRS for logic 1). All possible logic gates can be defined with two fundamental operations [32,33], namely the IMPLY (2-input 1-output operation, truth table in Figure 3d) and the FALSE (1-input 1-output operation always yielding logic 0). The COPY function (realized with IMPLY and FALSE) allows cascaded operations [12]. FALSE is executed by applying a negative voltage pulse to the RRAM (i.e., the “classical” reset operation), see Figure 3c. IMPLY between two logic values (stored in RRAMs P and Q) is executed by simultaneously applying two different positive voltage pulses at P (V_COND_) and Q (V_SET_), see Figure 3b. The result is stored in Q, while P must preserve its state. These requirements introduce several constraints that make the design space for the definition of V_SET_ and V_COND_ values very narrow [12,18], requiring a fine control down to the tens of mV, which is hard to achieve without huge overheads [12,18]. Further, the choice of R_G_ suffers from trade-offs [12]. Moreover, the repeated execution of IMPLY causes state drifts in P and Q, eventually causing bit corruption, as reported in [12,18]. Upon this, a refresh is needed that requires moving all data back and forth to a new memory location, wasting energy and time [12,18].

### 2.3. The “SIMPLY” Architecture

**Figure 4:**
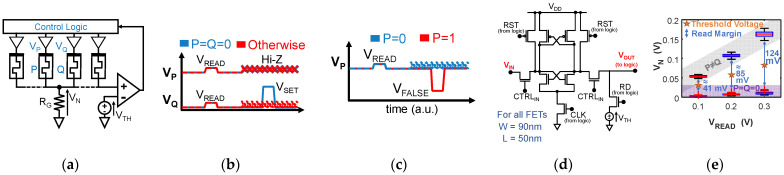
(**a**) Schematic of the SIMPLY architecture implemented on a linear RRAM array. (**b**) Pulses applied to the top electrodes of P (V_P_) and Q (V_Q_) to execute the IMPLY operation in SIMPLY architecture when the comparator detects P = Q = 0 (blue lines) and in all other cases (dashed red lines). (**c**) Pulse applied at the top electrode of P to perform the sFALSE P operation in SIMPLY architecture when P = 0 (dashed blue lines) and P = 1 (red lines). (**d**) Comparator implementation from [30] that is used in this work (45 nm technology [34]—MOSFETs W/L are shown). (**e**) Distribution of V_N_ (technology 3 [29]) resulting from circuit simulations including variability and RTN (50 trials) when P = Q = 0 (violet bands) and P ≠ Q (grey bands) for a the 2-SIMPLY operation at different V_READ_. The read margins (RM—blue arrows) and associated threshold voltages (V_TH_—orange stars) for the comparator are evidenced. Black whiskers indicate the extreme points of the distributions. Red crosses indicate outliers due to RTN. V_N_ when P = Q = 1 is always much higher than in all other cases (thus is not reported in these box plots).

**Figure 5:**
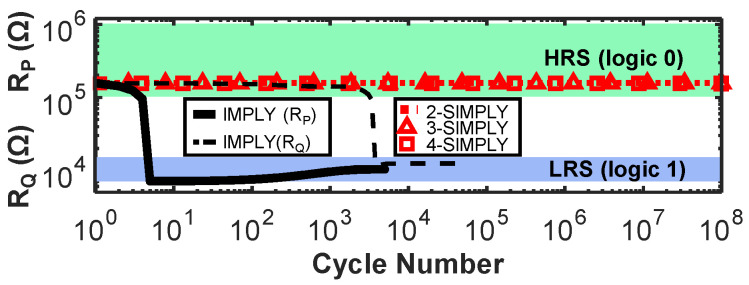
Degradation profiles of R_P_ (dashed black line) and R_Q_ (solid black line) over time during the repeated execution of IMPLY (technology 3 [29]). R_P_ degrades when repeating IMPLY for input combination P = Q = 0; R_Q_ degrades when repeating IMPLY for input combination P = 1 and Q = 0. The degradation depends on the initial values of R_Q_ and R_P_, (worst-cases for R_Q_ and R_P_ shown here, considering variability and RTN). Bit corruption occurs potentially after only 7 cycles (solid black line). No noticeable degradation occurs using SIMPLY (only worst-case reported) up to at least 10^8^ cycles, also when performing multi-input operations (i.e., *n*-SIMPLY when *n* = 2, 3, 4—dotted red line, red triangles, and red squares, respectively).

To overcome the issues of the traditional IMPLY architecture, SIMPLY was introduced in [20]. SIMPLY is based on the implication logic and originates from the observation that Q must change its state only when P = Q = 0 (case 1 of the truth table in Figure 3d). SIMPLY detects this condition by applying a small V_READ_ voltage pulse (200 mV in this work) to both P and Q, Figure 4b. The voltage at node N (V_N_, Figure 4a) is compared against a threshold (V_TH_) to determine if P = Q = 0. If so, the control logic which is equipped with tri-state buffers, as in the IMPLY architecture, pulses V_SET_ on Q, keeping the driver of P at high impedance; Figure 4b, blue lines. Otherwise, both P and Q are kept at high impedance; Figure 4b, dashed red lines. The condition P = Q = 0 is easily detectable since V_N_ is lower in this case than in all other cases, ensuring a sufficient margin (i.e., read margin—RM) also when considering RTN and variability, as shown in Figure 4e. Moreover, the RM increases with V_READ_, Figure 4e, which allows trading a higher RM (i.e., lower Bit Error Rate—BER) for power, thus setting precise parameter tradeoffs. SIMPLY requires no V_COND_ and no fine control of the voltage pulses, overcoming the tradeoff between V_SET_ and V_COND_ and the need for very precise voltage control. The problem of logic state degradation is virtually resolved, as only V_READ_ is applied to the devices that must retain their logic state. In fact, no degradation is observed up to (at least) 10^8^ cycles, reducing BER by at least 10^8^ compared to IMPLY, with no energy penalty (see Figure 5). Besides, SIMPLY needs significantly less energy as, for 3 out of 4 input combinations, only the two V_READ_ pulses are delivered instead of the much larger V_SET_ and V_COND_ pulses, Figure 4b. To ensure high energy efficiency, we use the low-power and compact comparator design from [30], implemented in a 45-nm technology [34] (Figure 4d), which consumes 8fJ per comparison when T is in the range 0 to 85 °C and V_DD_ is 2 V. The energy breakdown, including the comparator contribution, for all input combinations for IMPLY and SIMPLY, stresses the remarkable performance improvement, as SIMPLY reduces the energy per operation by 4× on average and up to 57× for the P = 0 Q = 1 input configuration, when considering technology 3 as an example (see Table 1). Depending on the RRAM technology, the energy consumption of the FALSE operation may be higher or lower than that of the IMPLYoperation (see Table 1 and Table 2). So, it is important to also reduce the energy of the FALSE. This can be easily done in the SIMPLY architecture, by preventing the use of the high |V_FALSE_| when a device is already in HRS. The state of the device is read using the same V_READ_ and comparator threshold used for the IMPLY operation, and the analog buffers are enabled by considering the inverted output of the comparator, see Figure 4c. This smart FALSE (sFALSE [30]) operation results in relevant energy savings (see Table 2). 

**Figure 6:**
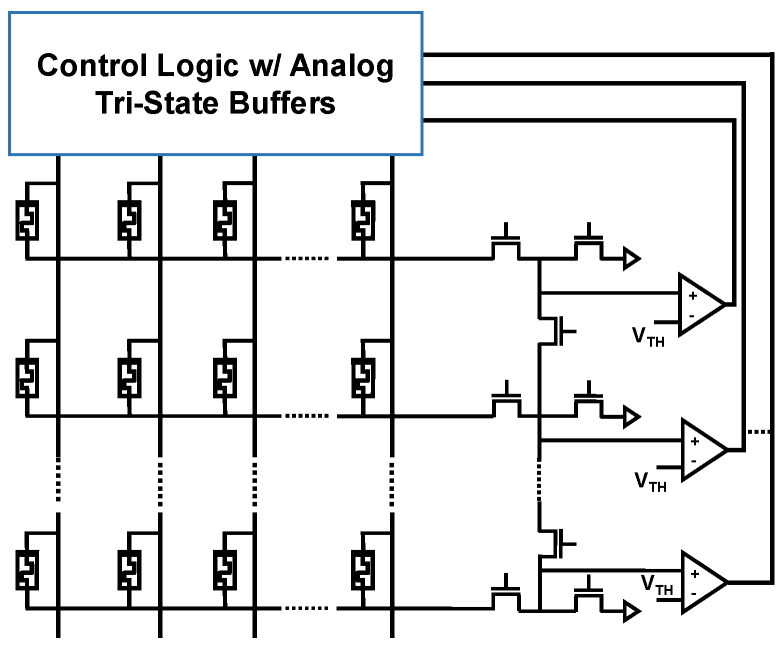
SIMPLY crossbar implementation. More than one comparator can be used to increase the computation parallelism.

In addition, both the traditional IMPLY and the SIMPLY architectures can be implemented in a crossbar array [12,30]. An example of the SIMPLY crossbar implementation is shown in Figure 6. FET devices in the array periphery are used to implement R_G,_ to connect adjacent columns on-demand to realize COPY between any two devices, and to enable specific columns of the array. The parallel computation of SIMPLY or sFALSE operations can be implemented by increasing the number of comparators in the architecture as discussed in [30] and shown in Figure 6.

## 3. Results and Discussion

### 3.1. Multi-Input Operations: “n-SIMPLY”

**Figure 7:**
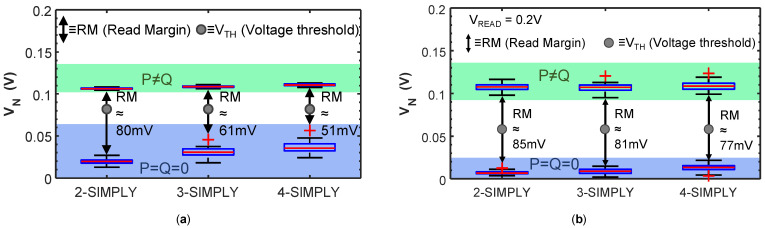
Distribution of the V_N_ voltage (**a**) experimentally evaluated with technology 4, and (**b**) simulated with the compact model calibrated on technology 3, for increasing number of input bits (i.e., n = 2, 3, 4), including variability (50 trials) when P = Q = 0 (blue bands) and P≠Q (green bands) for the *n*-SIMPLY operation (*n* = 2, 3, 4) at V_READ_ = 0.2 V. The read margins (RM—black arrows) and associated threshold voltages (V_TH_—grey circle) for the comparator are evidenced. Black whiskers indicate the extreme points of the distributions. Red crosses indicate outliers due to RTN. V_N_ when P = Q = 1 is always much higher than in all other cases (thus is not reported in these box plots).

In traditional static complementary CMOS logic gates, the computation of more complex operations (e.g., large fan-in operations), involves the use of additional transistors, therefore an increase in the required circuit area and complexity. In IMPLY-based architectures, the computation of complex operations instead translates to an increased number of steps of IMPLY and FALSE operations. Therefore, in these architectures, minimizing the number of steps is critical to improve the circuit performance. As suggested by theoretical works, the IMPLY logic can be extended to multi-input (>2) operations [32,35] by simultaneously applying V_SET_ to one device (Q) and V_COND_ to many devices (P, S, T, …). It is only recently that the three input IMPLY operation (i.e., equivalent to the OR(Q, NOR(P, S)) has been demonstrated by performing circuit simulations of a conventional IMPLY architecture using a physics-based compact model calibrated on a Pt/TaO_x_/Ta RRAM technology [21] and was shown to reduce the number of steps required for implementing a full adder. However, the reliability of the traditional IMPLY architecture worsens when the number of inputs of the IMPLY operation is increased [21]. Nevertheless, generalizing the IMPLY operation to more than three inputs allows further reducing the number of computing steps, by executing in a single step the more complex function $OR\left(Q,\;AND\left(\bar{P},\;\bar{S},\;\bar{T},\ldots \right)\right)=OR\left(Q,\;NOR\left(P,S,T,\ldots \right)\right)$, hereafter called n-IMPLY (n being the number of inputs) generalizing the IMPLY with 2 inputs (henceforth 2-IMPLY). Just like in 2-IMPLY, also in n-IMPLY Q must change its state to logic 1 only if all inputs are concurrently 0, that is exactly the condition detected by SIMPLY. So, n-IMPLY can be implemented in SIMPLY (henceforth n-SIMPLY) by applying V_READ_ to all n devices simultaneously with no architectural changes. We simulated the n-SIMPLY (with n = 2, 3, 4) for the 4 RRAM technologies explored in this work using the Cadence^®^ Virtuoso software and verified its correct functionality by comparing the circuit parameters and energy per operation (see Table 3), including variability and RTN. While the minimum and maximum energy per n-SIMPLY operation do not change considerably for increasing values of n, the RM decreases when the number of RRAMs read in parallel increases. Nevertheless, the RM with n = 2, 3, 4 was verified to be always large enough, experimentally on technology 4 (see Figure 7a), and by using simulations on the other three technologies, as shown in Figure 7b. Moreover, in all cases, no logic state degradation is observed (see red curves in Figure 5). 

### 3.2. Full Adder Implementation with n-SIMPLY

**Figure 8:**
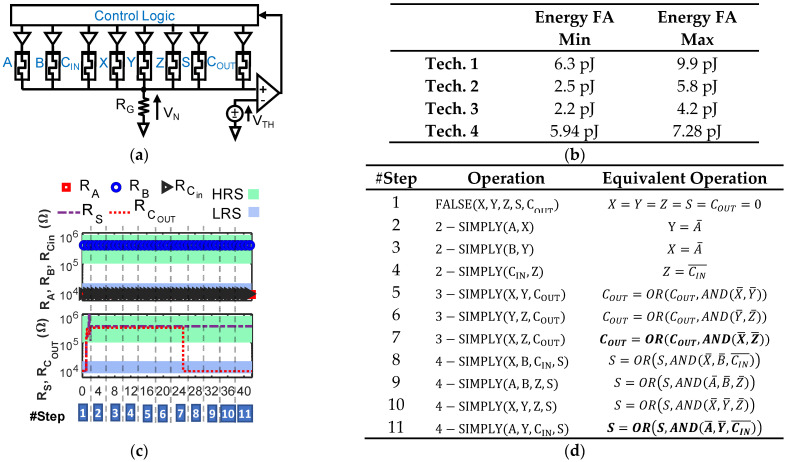
(**a**) Schematic of the proposed full adder (FA). A, B, C_IN_ are input devices (bits), X, Y, Z additional RRAMs, S and C_OUT_ output devices (bits). (**b**) FA total energy consumption for the 4 RRAM technologies used in this work, including variability and RTN, and considering all possible input bits’ combinations. (**c**) Resistance of devices A, B, C_IN_, S, and C_OUT_ during the FA sequence of operations (corresponding steps are reported in (**d**)) with A = 1, B = 0, C_IN_= 1 (technology 3). Input values keep unaltered. (**d**) List of operations required to compute the complete 1-bit full-addition.

Exploiting n-SIMPLY, we propose a novel implementation of a LIM 1-bit full adder (FA). The schematic of the array and the sequence of operations are shown in Figure 8a,d. We simulated the FA at 0.5 GHz (see the example in Figure 8c) using the 4 RRAM technologies and compared the total energy consumption (including the comparator and the effects of variability and RTN) in Figure 8b. Not surprisingly, technology 3 [29], which is the technology with the lowest current compliance (I_C_ = 100 µA, see Figure 1c), results in the lowest energy consumption. We considered the worst-case result (i.e., Energy FA max column in Figure 8b) obtained with technology 3 and benchmarked it in detail against existing LIM solutions in the literature and its CMOS counterpart in Table 4 and Table 5, respectively. Considering the worst-case energy contribution for each 1-bit FA operation results in an energy consumption overestimation, that, as a first-order approximation, provides enough room to account for additional energy dissipated by components in the peripheral circuitry that were not included in the circuit simulations (i.e., the analog tri-state buffers). The proposed FA outperforms all existing IMPLY-based LIM solutions (both simulation and experimental works [12,20,36,37,38]), in terms of the number of steps (delay) and energy consumption, using few devices and lowering the energy-delay product (EDP) by a factor >10^3^, bringing it much closer to the CMOS one. We compare the performance of the proposed solution vs. CMOS (with and without considering the VNB energy and time overhead) in Table 5 considering the parallel execution of 512 32-bit FA operations (simple ripple carry architecture), which entails 4kB data, which is the common memory page size [39].

The proposed solution strongly outperforms different CMOS FA solutions [40,41,42] (>10^6^ improvement in EDP) when VNB data exchange overhead [39] is included. Projections show that substantial improvements are obtained by using devices formed at lower I_C_ [43] and shorter pulses [44], as in Table 5, achieving the performance of CMOS gates without the huge VNB penalty (up to a ≈2.4 × 10^10^ improvement in EDP as compared to a CMOS FA that suffers from VNB). 

### 3.3. Binarized Neural Networks Applications

As the adoption of deep learning and neural networks is becoming more and more pervasive, the demand for energy efficient hardware accelerators is rapidly growing. When considering neural networks, the most effective way to improve energy efficiency is to avoid the VNB by performing computations in-memory [45,46,47,48]. A common approach exploits resistive memory crossbar arrays to implement in analog the vector matrix multiplication in a single step [45]. However, reliability issues affecting RRAM devices, such as the large cycle-to-cycle variability, limit the number of bits that can be reliably stored in a single device [49] thus suggesting that low-bit precision neural networks are a more suitable solution for the current state of the art RRAM devices. The extreme case of low-bit precision neural networks are BNNs, which have been shown to retain high classification accuracy despite the use of single-bit neuron weights and activations [5,50,51]. While the in-memory computation of the analog vector matrix multiplication is extremely attractive [6,7,50,52], it has some limitations and tradeoffs. In fact, this approach lacks the possibility to reconfigure the type of operation to be computed in-memory.

Since neurons in BNNs perform logic operations, the BNNs inference can be implemented in the SIMPLY architecture, as shown in [30] where a BNN was implemented using the 2-SIMPLY. Compared to an analog implementation, the SIMPLY architecture trades reconfigurability for larger computation latency. To address the higher latency issue, the multi-input IMPLY operation can be used as discussed below. 

**Figure 9:**
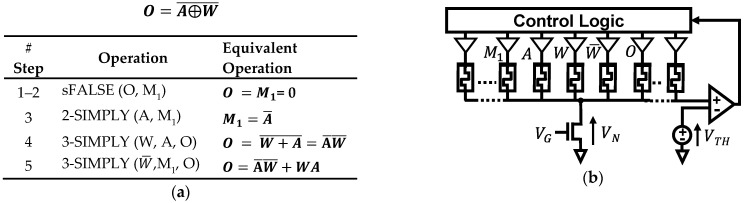
(**a**) Sequence of IMPLY and FALSE operations required for implementing the XNOR operation using n-SIMPLY. While 9 steps and 2 support devices are needed when using only 2-SIMPLY [30], the increased parallelism provided by n-SIMPLY reduces to 5 the number of computing steps, and only 1 support device is needed. (**b**) Circuit implementation of the XNOR operation on the SIMPLY architecture.

**Figure 10:**
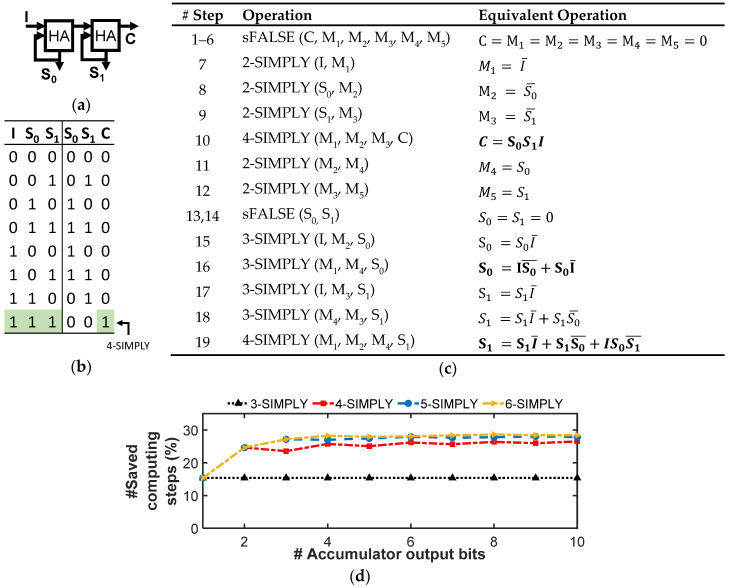
n-SIMPLY-based accumulator for implementing the popcounting operation used in BNNs. (**a**) Two serially connected half-adders (HAs) used to implement the popcount operation. (**b**) Truth table of the sequence of two serially connected half-adders (HAs). The carry-out (C) of the complete operation is computed in a single step by using a 4-SIMPLY. (**c**) Sequence of IMPLY and FALSE operations implementing the operation described by the truth table in (**b**). The use of 4-SIMPLY reduces the number of computing steps from 26 (when using only the 2-SIMPLY [30]) to 19. (**d**) Percentage of saved computing steps for increasing number of output bits and increasing parallelism with respect to the baseline 2-SIMPLY implementation from [30]. Most computing steps are saved by using the 3-SIMPLY and up to the 4-SIMPLY operations, while further increasing the parallelism of the SIMPLY operation results in a limited improvement.

As shown in [30], in BNNs, multiplications between the inputs and each neuron’s weights are implemented with bitwise XNOR operations, the accumulation as the popcount operation, and the activation as a comparison with a trained threshold. In addition, batch normalization layers can be implemented with full adders for which we have already discussed the optimized n-SIMPLY implementation. The effectiveness of the multi-input IMPLY operation in reducing the number of computing steps is analyzed for each logic function since it is strongly dependent on the associated specific sequence of operations. As shown in Figure 9, by using the 3-SIMPLY the XNOR operation is computed in 5 steps while using a single additional device, instead of requiring 9 steps and 2 additional support devices when using only the 2-SIMPLY. For the popcount and activation function operations, different optimization strategies that minimize the number of computing steps for different fan-in (i.e., n) of the n-SIMPLY operation can be employed. As shown in [30], the accumulator can be implemented as a chain of log_2_(#input bits) 1-bit half adders (HAs) where the first HA receives in input the bit that needs to be accumulated and its current output (i.e., S_0_ in Figure 10a), while the following HAs receive at their inputs the carry-out from the previous HA and their current output (i.e., as in in Figure 10a but also for more than two HAs). When accumulating a sequence of bits, all the bits are input sequentially to the HA representing the LSB. The operations of the subsequent HAs are performed only after a number of input bits equal to their relative bit position have been summed (e.g., HA with bit position 3 is activated only after 2^3^ input bits have been summed). Thus, the length of the HA chain grows as more input bits have been summed. By using the 3-SIMPLY operation, the number of steps for each HA operation is reduced from 13 to 11, see Supplementary Figure S1. An effective strategy that can further reduce the total number of computing steps involves the use of the n-SIMPLY operation to compute the equivalent logic function resulting from a sequence of more than one 1-bit HAs. Thereby, the equivalent carry-out of a sequence of more than 1 HA (output C in Figure 10a), can be computed in a single step by exploiting the n-SIMPLY operation (with n = #HAs + 2). For instance, the carry-out can be computed with a 4-SIMPLY or a 5-SIMPLY when a sequence of 2 or 3 HAs is considered, respectively. Moreover, the number of intermediate computing steps required to calculate the output bits S_i_ (see Figure 10a) is reduced using the n-SIMPLY, as shown in Figure 10b and Supplementary Figure S2–S4, where the sequence of operations computing the result of a sequence of 2, 3, and 4 HAs are reported. Specifically, 19 (see Figure 10c), 28 (see Supplementary Figure S3), and 37 computing steps (see Supplementary Figure S4), for a sequence of 2, 3, and 4 HAs, respectively, are required instead of 26, 39, and 52 steps when using only the 2-SIMPLY. The overall number of computing steps scales rapidly with the number of output bits, but the use of n-SIMPLY with n up to 3 (i.e., only the chain of 2 HAs is optimized) provides a saving of around 15% of steps while for n > 3 leads to a step saving above 25%. However, most of the reduction in the number of computing steps is achieved when using n-SIMPLY with n up to 4 while the use of n > 4 provides only a limited improvement, as shown in Figure 10d, that does not justify the risk of reducing the reliability of the circuit due to the smaller available RMs.

**Figure 11:**
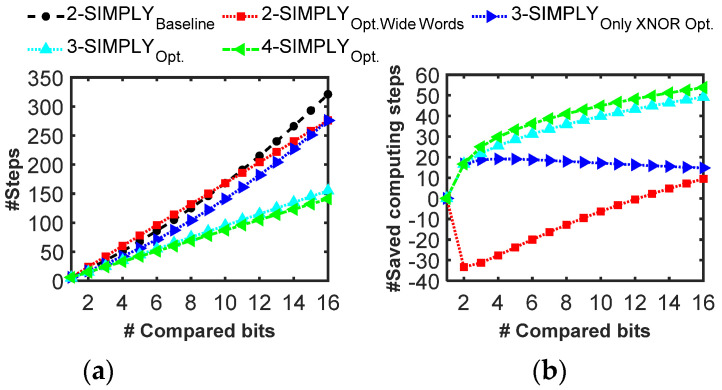
Analysis of the comparator implementation for increasing n of the n-SIMPLY operation and number of compared bits. Both the total number of computing steps and the percentage of saved computing steps are reported in (**a**,**b**), respectively. The use of optimized sequences for the 3-SIMPLY (i.e., 3-SIMPLY_Opt._) and 4-SIMPLY (i.e., 4-SIMPLY_Opt._) operations considerably reduces the number of computing steps.

For the activation (i.e., comparison with a trained threshold), we improved the 2-SIMPLY implementation from [30], by removing unnecessary computing steps. The resulting sequence of computing steps can be built using the flowchart reported in Supplementary Figure S5, which consists in the computation of a bitwise XNOR between the input and the trained threshold, and additional AND/OR operations. By following this method, the number of computing steps scales exponentially with the number of compared input bits (i.e., 2-SIMPLY_baseline_ in Figure 11). Thus, we also consider an alternative approach in which the number of computing steps scales linearly with the number of input bits, reducing the number of computing steps when more than 9 bits are compared (see 2-SIMPLY_Opt.WideWords_ in Figure 11 and Supplementary Figure S6). The same two approaches can be optimized with the 3-SIMPLY operation (see 3-SIMPLY_OnlyXNOROpt._ and 3-SIMPLY_Opt._) providing a considerable reduction in the number of computing steps (see Supplementary Figures S7 and S8). While the use of the 4-SIMPLY operation enables additional step savings (see 4-SIMPLY_Opt._ in Figure 11 and Supplementary Figure S9), n-SIMPLY with larger n values would not provide any additional step reduction. 

Thus, some applications, such as the BNN inference, can largely benefit from a limited increase in n-SIMPLY operation parallelism. In fact, most of the step reduction for a 1-bit FA, the bitwise XNOR operation, the popcounting operation, and the activation function are achieved by using only up to the 4-SIMPLY operation. Further increasing the parallelism of the n-SIMPLY operation would not provide relevant additional energy savings while causing a reduction in the available RM, thus potentially reducing the circuit reliability, and increasing the instruction set complexity. 

To benchmark the latency improvement brought by the n-SIMPLY operation when performing an inference task, we consider the results of the 2-SIMPLY implementation of a BNN reported in [30], which consists of a single fully connected hidden layer with 1000 neurons performing a handwritten digit recognition task, achieving an accuracy of 91.4%. The use of n-SIMPLY reduces the number of computing steps from 190,657 to 135,539, therefore resulting in a remarkable 29% latency reduction leading to a 542 µs inference time at 0.5 GHz, which further highlights the advantages of the SIMPLY architecture as a solution for ultra-low power devices for edge computing applications.

Additionally, unlike conventional IMPLY architectures, SIMPLY is not constrained to employ bipolar RRAM devices [18] and can use unipolar RRAM devices as well as other memory technologies such as phase change memories (PCM) [53], ferroelectric FETs (FeFETs) [54], ferroelectric tunnel junctions (FTJs) [54], and virtually any 2- or 3-terminal non-volatile memory. Therefore, device/circuit co-design strategies can be implemented easily to further improve performance. For instance, memory technologies with ultra-low power consumption like FeFETs and FTJs [54] may be targeted to improve power consumption. Further, devices with higher endurance than most RRAM technologies, such as PCM [54] and STT-MRAM [53], could be evaluated on the SIMPLY architecture.

## 4. Conclusions

In this work, we presented the advantages of the multi-input IMPLY operation performed on SIMPLY (n-SIMPLY), a new LIM edge computing architecture that overcomes all the relevant issues of traditional IMPLY solutions. The advantages of multi-input SIMPLY operations were analyzed in detail using a comprehensive physics-based RRAM compact model calibrated on three different RRAM technologies in the literature and validated by the experimental analysis carried out on fabricated devices. The performance comparison of a 1-bit full adder based on n-SIMPLY and state-of-the-art LIM alternatives present in the literature shows that n-SIMPLY allows realizing a LIM solution that approaches the performance of CMOS gates while bypassing VNB, with a huge improvement in BER (by a factor of at least 10^8^) and EDP (up to a factor 10^10^). Moreover, we analyze the advantages brought by n-SIMPLY on a BNN inference task and show that it enables a latency reduction of 29% with respect to previous studies.

## Figures and Tables

**Table 1:** Energy consumption comparison of the 2-IMPLY operation in IMPLY and SIMPLY architectures with technology 3. The use of SIMPLY enables the use of a lower V_SET_ voltage (V_SET, IMPLY_ = 3.2 V, V_SET, SIMPLY_ = 3 V). In all cases, 1ns voltage pulses are considered (t_2-IMPLY_ = 2·t_PULSE_, t_2-SIMPLY_ = 4·t_PULSE_), V_READ_ = 0.2 V, the effects of variability, RTN, and comparator energy overhead are included. Energy consumption data are from [30].

Input Combination	Energy 2-IMPLY (Min–Avg–Max)	Energy 2-SIMPLY (Min–Avg–Max)	2-SIMPLY Average Energy Improvement
P = 0 Q = 0	533–626–669 fJ	498–532–557 fJ	1.2
P = 0 Q = 1	657–672–691 fJ	11.7–11.8–11.9 fJ	57
P = 1 Q = 0	251–266–287 fJ	11.7–11.8–11.9 fJ	23
P = 1 Q = 1	660–678–699 fJ	12.6–12.6–12.6 fJ	54

**Table 2:** Energy consumption of FALSE operation in IMPLY and SIMPLY architectures for different RRAM technologies.

Parameters	Technology 1	Technology 2	Technology 3	Technology 4
Energy FALSE (min-avg-max) *	294–541–953 fJ	160–660–1162 fJ	90–278–492 fJ	463–736–1471 fJ
Energy sFALSE (min-avg-max) *	17–355–932 fJ	10–406–1224 fJ	9–175–492 fJ	23.8–523–1446 fJ

* The comparator overhead, variability, and RTN are included in the simulations. The average (avg) energy per operation is determined considering equiprobable initial conditions.

**Table 3:** Circuit parameters and energy consumption of n-SIMPLY operation (n = 2, 3, 4) with t_pulse_ = 1 ns.

Parameters	Tech. 1	Tech. 2	Tech. 3	Tech. 4
R_G_	2 kΩ	6 kΩ	10 kΩ	1.5 kΩ
V_SET_	1.9 V	1.7 V	3 V	1 V
V_READ_	0.2 V	0.2 V	0.2 V	0.2 V
V_FALSE_	−1.55 V	−2 V	−2.8 V	−1.54 V
Energy 2-SIMPLY (min–max) *	29 fJ–2.1 pJ	13 fJ–370 fJ	12 fJ–571 fJ	35.4–462 fJ
Energy 3-SIMPLY (min–max) *	30 fJ–2.2 pJ	18 fJ–371 fJ	13 fJ–568 fJ	32.6–462 fJ
Energy 4-SIMPLY (min–max) *	29 fJ–2.2 pJ	12 fJ–380 fJ	12 fJ–567 fJ	38.7–465 fJ

* The comparator overhead, variability, and RTN are included in the simulations by repeating the simulation 50 times for each input configuration.

**Table 4:** Detailed comparison among the proposed and existing FAs in the literature. SIMPLY, IMPLY, and Hybrid-CMOS LiM solutions (both experimental and simulation works).

Author(s)	Type of Logic (exp/sim)	Physics-Based Model	# Devices	Feasible in Crossbar	Energy * (Estimated/Reported)	# Elementary Steps	Delay * (Estimated/Reported)	Retains Input Values	Endurance before Refresh **
Siemon et al. [21]	IMPLY (sim)	YES	8 RRAM	YES	202 pJ (estimated)	19	3.61 μs (estimated)	NO	?
Lehtonen et al. [32]	IMPLY (sim)	NO	8 RRAM	YES	-	136	-	YES	?
Kvatinsky et al. [12]	IMPLY (sim)	NO	9 RRAM	YES	-	23	9.1 μs (estimated)	NO	≈300 up to 10^5^ (trades with energy)
Kvatinsky et al. [12]	IMPLY (sim)	NO	6 RRAM	YES	-	29	11.5 μs (estimated)	NO	≈300 up to 10^5^ (trades with energy)
Cheng et al. [36]	IMPLY (exp)	-	8 RRAM	YES	19.5 pJ (reported)	27	54 μs (reported)	NO	?
Zanotti et al. [20]	IMPLY (sim)	YES	9 RRAM	YES	6.4 nJ (reported)	43	345 ns (reported)	YES	67
Zanotti et al. [20]	IMPLY (sim)	YES	8 RRAM	YES	518 pJ	28	560 ns	YES	≈ 30
Zanotti et al. [20]	SIMPLY (sim)	YES	8 RRAM	YES	172 pJ	28	920 ns (reported at 0.05GHz)	YES	>4.5·10^6^ (no energy trade-off)
**This Work**	**n-SIMPLY (sim)**	**YES**	**8 RRAM**	**YES**	**4.2 pJ**	**11**	**42 ns**	**YES**	**>4.5·10^6^** **(no energy trade-off)**
Junsangsri et al. [37]	CMOS LIM (sim)	YES (FET) NO (RRAM)	41 FET + 4 RRAM	NO	2.2 fJ (reported—excludes RRAM energy)	-	52 ps (reported—excludes RRAM delay)	YES	? (Limited by FET Reliability)

* Reported means that the value was explicitly reported by the authors in the paper. Estimated means that the corresponding value has been inferred from data in the paper, although not explicitly reported by the authors. ** This information can be trusted only if a physics-based model is used.

**Table 5:** Comparison between the proposed FA and a CMOS FA when executing 512 parallel 32-bit FA operations (4 kB data).

	Number of Computing Devices	Energy	Delay	Energy-Delay Product (EDP)	EDP Improvement Normalized to CMOS w/VNB
CMOS w/ VNB *	163840–458752 FET	≈85.5 µJ	≈ 2.6 ms	≈ 2.2 × 10^−7^ J·s	1
CMOS w/o VNB **	163840–458752 FET	≈8.8 × 10^−7^–107 nJ	≈ 0.2–1.2 × 10^5^ ns	≈ 2.5 × 10^−25^–1.4 × 10^−11^ J·s	1.6 × 10^4^–8.9 × 10^17^
This Work ***	**18944 RRAM**	**≈68.8 nJ**	**≈1.3 µs**	**≈8.9 × 10^−14^ J·s**	**2.4 × 10^6^**
**Projections** I_C_ = 10 nA f = 1 GHz ****	**18944 RRAM**	**≈6.88 pJ**	**≈ 0.65 µs**	**≈4.5 × 10^−18^ J·s**	**4.9 × 10^10^**

*, **, estimates with (w/) and without (w/o) including the VNB energy and delay overhead for reading and writing 4 kB data [39]. ** CMOS FA performances were estimated projecting the time and energies for different 1-bit FA schemes and different CMOS technologies (i.e., 0.18 µm, 45 nm, and 10 nm) from [40,41,42] combined in a ripple carry configuration. ***, **** Worst-case energy estimates considering the maximum energy for each single 1-bit FA operation using Technology 3, see Figure 8b. This results in an energy overestimation that approximately accounts for additional energy overhead possibly introduced by the peripheral circuit (i.e., analog tri-state buffers contributions). **** Projections using optimized devices with I_C_ = 10 nA [43] and f = 1 GHz [44].

## Supplementary Materials

The following are available online at https://www.mdpi.com/article/10.3390/mi12101243/s1, Figure S1: 1-bit HA sequence of computing steps using (a) 2-SIMPLY only and, (b) up to 3-SIMPLY. The use of 3-SIMPLY reduces the number of computing steps from 13 to 11. Figure S2. 4-SIMPLY-based implementation of two concatenated 1-bit HAs that are used to implement the popcounting operation for BNNs. (a) Sketch of the two serially connected half-adders (HAs). (b) Sequence of IMPLY and FALSE operations implementing the result of the operation. The use of 4-SIMPLY reduces the number of computing steps from 26 (when using only the 2-SIMPLY Figure S1a) to 19. Figure S3. 5-SIMPLY-based implementation of three concatenated 1-bit HAs that are used to implement the popcounting operation for BNNs. (a) Sketch of the three serially connected half-adders (HAs). (b) Sequence of IMPLY and FALSE operations implementing the result of the operation. The use of 5-SIMPLY reduces the number of computing steps from 39 (when using only the 2-SIMPLY Figure S1a) to 28. Figure S4. 6-SIMPLY-based implementation of four concatenated 1-bit HAs that are used to implement the popcounting operation for BNNs. (a) Sketch of the four serially connected half-adders (HAs). (b) Sequence of IMPLY and FALSE operations implementing the result of the operation. The use of 6-SIMPLY reduces the number of computing steps from 52 (when using only the 2-SIMPLY Figure S1a) to 37. Figure S5. Flowchart building the sequence of computing steps required for implementing the activation function of a BNN neuron, i.e., the comparison with a threshold (TH), when using the 2-SIMPLY operation (i.e., 2-SIMPLY-baseline in Figure 11). The formula for the comparison operation is reported together with the scaling of the number of computing steps (i.e., #steps) as a function of the number of compared input bits (n). Figure S6. Flowchart building the sequence of computing steps optimized for large number of input bits, that is used to implement the activation function of a BNN neuron, i.e., the comparison with a threshold (TH), when using the 2-SIMPLY operation (i.e., 2-SIMPLY Opt. Wide Words in Figure 11). The formula for the comparison operation is reported together with the scaling of the number of computing steps (i.e., #steps) as a function of the number of compared input bits (n). Figure S7. Flowchart building the sequence of computing steps that is used to implement the activation function of a BNN neuron, i.e., the comparison with a threshold (TH), when using the 3-SIMPLY operation and optimizing only the steps required for computing the intermediate XNOR operations (i.e., 3-SIMPLY Only XNOR in Figure 11). The formula for the comparison operation is reported together with the scaling of the number of computing steps (i.e., #steps) as a function of the number of compared input bits (n). Figure S8. Flowchart building the optimized sequence of computing steps that is used to implement the activation function of a BNN neuron, i.e., the comparison with a threshold (TH), when using the 3-SIMPLY operation (i.e., 3-SIMPLY Opt. in Figure 11). The formula for the comparison operation is reported together with the scaling of the number of computing steps (i.e., #steps) as a function of the number of compared input bits (n). Figure S9. Flowchart building the optimized sequence of computing steps that is used to implement the activation function of a BNN neuron, i.e., the comparison with a threshold (TH), when using the 4-SIMPLY operation (i.e., 4-SIMPLY Opt. in Figure 11). The formula for the comparison operation is reported together with the scaling of the number of computing steps (i.e., #steps) as a function of the number of compared input bits (n).
