# Supplementary material for: Multi-Input Logic-in-Memory for Ultra-Low Power Non-Von Neumann Computing
Source: Micromachines (Basel). 2021 Oct 14;12(10):1243. doi: 10.3390/mi12101243 (PMC8538894; doi:10.3390/mi12101243)
Supplement: Supplementary file 1 [file micromachines-12-01243-s001.zip › micromachines-1394142-supplementary.pdf]

Supplementary Material

Multi-input Logic-in-Memory for Ultra-Low Power non-von Neumann Computing

Tommaso Zanotti [1,*], Paolo Pavan [1] and Francesco Maria Puglisi [1]

[1] Dipartimento di Ingegneria "Enzo Ferrari", Università di Modena e Reggio Emilia, Via P. Vivarelli 10/1, 6 41125 Modena, Italy; francescomaria.puglisi@unimore.it (F.M.P.); paolo.pavan@unimore.it (P.P.)

* Correspondence: tommaso.zanotti@unimore.it;

# Step	Operation	Equivalent Operation
1-4	FALSE (C, M_1, M_2, M_3)	$C = M_1 = M_2 = M_3 = 0$
5	2-SIMPLY (I, M_1)	$M_1 = \bar{I}$
6	2-SIMPLY (S_0, M_2)	$M_2 = \bar{S_0}$
7	2-SIMPLY (S_0, M_1)	$M_1 = \overline{S_0 I}$
8	2-SIMPLY (M_2, I)	$I = \overline{\bar{S_0}\bar{I}}$
9	2-SIMPLY (M_1, M_3)	$M_3 = S_0 I$
10	2-SIMPLY (I, M_3)	$M_3 = S_0 I + \bar{S_0}\bar{I}$
11	FALSE (S_0)	$S_0 = 0$
12	2-SIMPLY (M_3, S_0)	$\boldsymbol{S_0 = S_0\bar{I} + \overline{S_0} I}$
13	2-SIMPLY (M_1, C)	$\boldsymbol{C = S_0 I}$

(**a**)

# Step	Operation	Equivalent Operation
1-4	sFALSE (C, M_1, M_2, M_3)	$C = M_1 = M_2 = M_3 = 0$
5	2-SIMPLY (I, M_1)	$M_1 = \bar{I}$
6	2-SIMPLY (S_0, M_2)	$M_2 = \bar{S_0}$
7	3-SIMPLY (M_1, M_2, C)	$\boldsymbol{C = S_0 I}$
8	2-SIMPLY (M_2, M_3)	$M_3 = S_0$
9	FALSE (S_0)	$S_0 = 0$
10	2-SIMPLY (M_2, I, S_0)	$S_0 = S_0\bar{I}$
11	2-SIMPLY (M_3, M_1, S_0)	$\mathbf{S_0 = I\overline{S_0} + S_0\bar{I}}$

(**b**)

Figure S1. 1-bit HA sequence of computing steps using **(a)** 2-SIMPLY only and, **(b)** up to 3-SIMPLY. The use of 3-SIMPLY reduces the number of computing steps from 13 to 11.

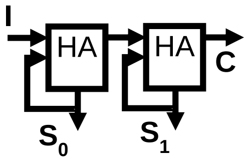

# Step	Operation	Equivalent Operation
1-6	sFALSE (C, M_1, M_2, M_3, M_4, M_5)	$C = M_1 = M_2 = M_3 = M_4 = M_5 = 0$
7	2-SIMPLY (I, M_1)	$M_1 = \bar{I}$
8	2-SIMPLY (S_0, M_2)	$M_2 = \bar{S_0}$
9	2-SIMPLY (S_1, M_3)	$M_3 = \bar{S_1}$
10	4-SIMPLY (M_1, M_2, M_3, C)	$\boldsymbol{C = S_0 S_1 I}$
11	2-SIMPLY (M_2, M_4)	$M_4 = S_0$
12	2-SIMPLY (M_3, M_5)	$M_5 = S_1$
13-14	sFALSE (S_0, S_1)	$S_0 = S_1 = 0$
15	3-SIMPLY (I, M_2, S_0)	$S_0 = \bar{I} S_0$
16	3-SIMPLY (M_1, M_4, S_0)	$\boldsymbol{S_0 = I\overline{S_0} + S_0\bar{I}}$
17	3-SIMPLY (I, M_3, S_1)	$S_1 = S_1\bar{I}$
18	3-SIMPLY (M_4, M_3, S_1)	$\boldsymbol{S_1 = S_1\bar{I} + S_1\overline{S_0}}$
19	4-SIMPLY (M_1, M_2, M_4, S_1)	$\boldsymbol{S_1 = S_1\bar{I} + S_1\overline{S_0} + IS_0\overline{S_1}}$

(**a**) **(b)**

Figure S2. 4-SIMPLY-based implementation of two concatenated 1-bit HAs that are used to implement the popcounting operation for BNNs. (**a**) Sketch of the two serially connected half-adders (HAs). (**b**) Sequence of IMPLY and FALSE operations implementing the result of the operation. The use of 4-SIMPLY reduces the number of computing steps from 26 (when using only the 2-SIMPLY Figure S1a) to 19.

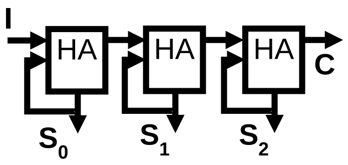

# Step	Operation	Equivalent Operation
1-8	sFALSE (C, M_1, M_2, M_3, M_4, M_5, M_6, M_7)	$C = M_1 = M_2 = M_3 = M_4 = M_5 = M_6 = M_7 = 0$
9	2-SIMPLY (I, M_1)	$M_1 = \bar{I}$
10	2-SIMPLY (S_0, M_2)	$M_2 = \bar{S_0}$
11	2-SIMPLY (S_1, M_3)	$M_3 = \bar{S_1}$
12	2-SIMPLY (S_2, M_6)	$M_6 = \bar{S_2}$
13	5-SIMPLY (M_1, M_2, M_3, M_6, C)	$\boldsymbol{C = S_0 S_1 S_2 I}$
14	2-SIMPLY (M_2, M_4)	$M_4 = S_0$
15	2-SIMPLY (M_3, M_5)	$M_5 = S_1$
16	2-SIMPLY (M_6, M_7)	$M_7 = S_2$
17-19	sFALSE (S_0, S_1, S_2)	$S_0 = S_1 = S_2 = 0$
20	3-SIMPLY (I, M_2, S_0)	$S_0 = S_0 I$
21	3-SIMPLY (M_1, M_4, S_0)	$\mathbf{S_0 = I\overline{S_0} + S_0\bar{I}}$
22	3-SIMPLY (I, M_3, S_1)	$S_1 = S_1 \bar{I}$
23	3-SIMPLY (M_4, M_3, S_1)	$\boldsymbol{S_1 = S_1 \bar{I} + S_1 \overline{S_0}}$
24	4-SIMPLY (M_1, M_2, M_4, S_1)	$\boldsymbol{S_1 = S_1 \bar{I} + S_1 \overline{S_0} + I S_0 \overline{S_1}}$
25	3-SIMPLY (M_5, M_2, S_2)	$S_2 = S_0 \overline{S_1}$
26	3-SIMPLY (I, M_6, S_2)	$S_2 = S_0 \overline{S_1} + \bar{I} S_2$
27	3-SIMPLY (M_6, M_4, S_2)	$S_2 = S_0 \overline{S_1} + \bar{I} S_2 + S_2 \overline{S_0}$
28	5-SIMPLY (M_1, M_2, M_3, M_7, S_2)	$\boldsymbol{S_2 = S_0 \overline{S_1} + \bar{I} S_2 + S_2 \overline{S_0} + I S_0 S_1 \overline{S_2}}$

(a) **(b)**

Figure S3. 5-SIMPLY-based implementation of three concatenated 1-bit HAs that are used to implement the popcounting operation for BNNs. (**a**) Sketch of the three serially connected half-adders (HAs). (**b**) Sequence of IMPLY and FALSE operations implementing the result of the operation. The use of 5-SIMPLY reduces the number of computing steps from 39 (when using only the 2-SIMPLY Figure S1a) to 28.

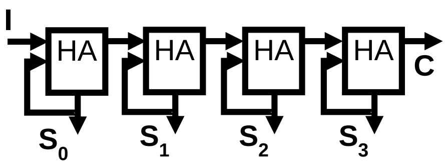

(**a**)

# Step	Operation	Equivalent Operation
1-10	sFALSE (C, M_1, M_2, M_3, M_4, M_5,M_6,M_7,M_8,M_9)	$C = M_1 = M_2 = M_3 = M_4 = M_5 = M_6 = M_7 = M_8 = M_9 = 0$
11	2-SIMPLY (I, M_1)	$M_1 = \bar{I}$
12	2-SIMPLY (S_0, M_2)	$M_2 = \bar{S_0}$
13	2-SIMPLY (S_1, M_3)	$M_3 = \bar{S_1}$
14	2-SIMPLY (S_2, M_6)	$M_6 = \bar{S_2}$
15	2-SIMPLY (S_3, M_8)	$M_8 = \bar{S_3}$
16	6-SIMPLY (M_8, M_1, M_2, M_3, M_6, C)	$\boldsymbol{C = S_0 S_1 S_2 S_3 I}$
17	2-SIMPLY (M_2, M_4)	$M_4 = S_0$
18	2-SIMPLY (M_3, M_5)	$M_5 = S_1$
19	2-SIMPLY (M_6, M_7)	$M_7 = S_2$
20	2-SIMPLY (M_8, M_9)	$M_9 = S_3$
21-24	sFALSE (S_0, S_1, S_2, S_3)	$S_0 = S_1 = S_2 = S_3 = 0$
25	3-SIMPLY (I, M_2, S_0)	$S_0 = S_0 I$
26	3-SIMPLY (M_1, M_4, S_0)	$\boldsymbol{S_0 = I\overline{S_0} + S_0\bar{I}}$
27	3-SIMPLY (I, M_3, S_1)	$S_1 = S_1\bar{I}$
28	3-SIMPLY (M_4, M_3, S_1)	$\boldsymbol{S_1 = S_1\bar{I} + S_1\overline{S_0}}$
29	4-SIMPLY (M_1, M_2, M_4, S_1)	$\boldsymbol{S_1 = S_1\bar{I} + S_1\overline{S_0} + I S_0\overline{S_1}}$
30	3-SIMPLY (M_5, M_2, S_2)	$S_2 = S_0\overline{S_1}$
31	3-SIMPLY (I, M_6, S_2)	$S_2 = S_0\overline{S_1} + \bar{I}S_2$
32	3-SIMPLY (M_6, M_4, S_2)	$S_2 = S_0\overline{S_1} + \bar{I}S_2 + S_2\overline{S_0}$
33	5-SIMPLY (M_1, M_2, M_3, M_7, S_2)	$\boldsymbol{S_2 = S_0\overline{S_1} + \bar{I}S_2 + S_2\overline{S_0} + I S_0 S_1\overline{S_2}}$
34	3-SIMPLY (I, M_8, S_3)	$S_3 = S_3\bar{I}$
35	4-SIMPLY (M_1, M_7, M_8, S_3)	$S_3 = S_3\bar{I} + I S_3\overline{S_2}$
36	4-SIMPLY (M_1, M_4, M_8, S_3)	$S_3 = S_3\bar{I} + I S_3\overline{S_2} + I S_3\overline{S_0}$
37	4-SIMPLY (M_1, M_5, M_8, S_3)	$\boldsymbol{S_3 = S_3\bar{I} + I S_3\overline{S_2} + I S_3\overline{S_0} + S_3\bar{I} + I S_3\overline{S_2} + I S_3\overline{S_1}}$

(**b**)

Figure S4. 6-SIMPLY-based implementation of four concatenated 1-bit HAs that are used to implement the popcounting operation for BNNs. (**a**) Sketch of the four serially connected half-adders (HAs). (**b**) Sequence of IMPLY and FALSE operations implementing the result of the operation. The use of 6-SIMPLY reduces the number of computing steps from 52 (when using only the 2-SIMPLY Figure S1a) to 37.

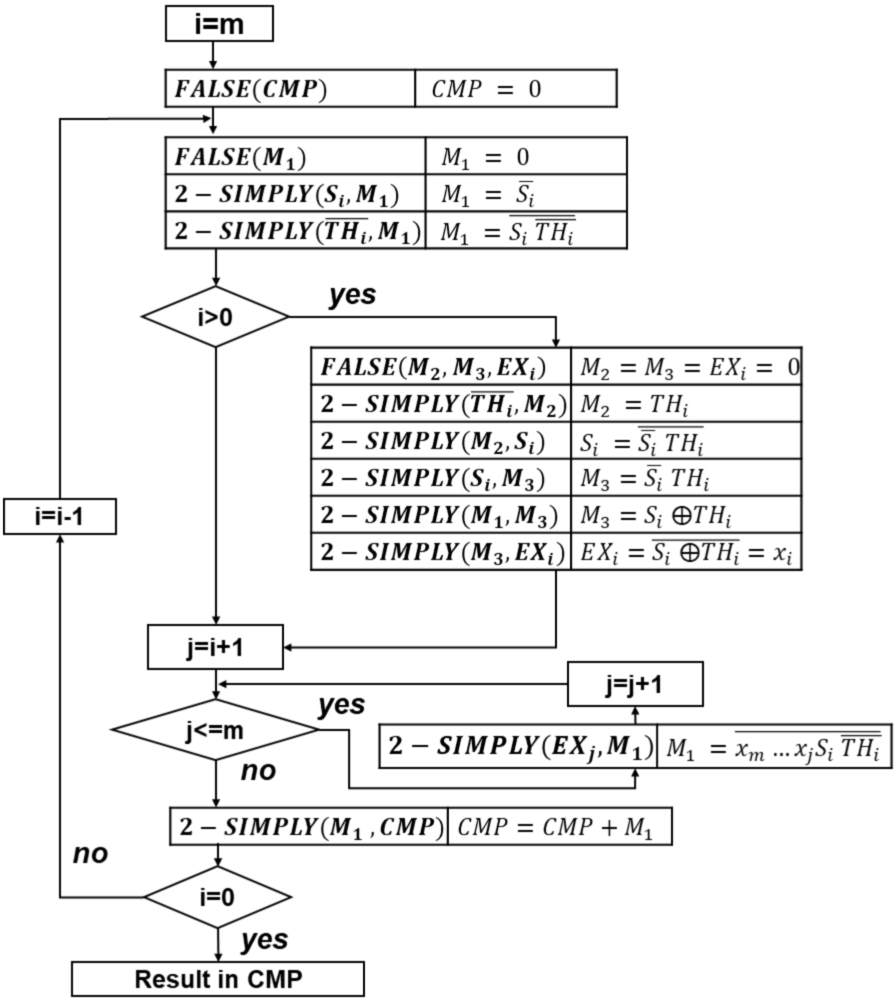

$$CMP = S_m\overline{TH_m} + x_m S_{m\_1}\overline{TH_{m-1}} + \cdots + x_m \cdot \ldots \cdot x_1 S_0 \overline{TH_0}$$

where $x_m = \overline{S_m \oplus TH_m}$

$n = m + 1$

$\boldsymbol{\#Steps = 0.5\, n^2 + 11.5n - 7}$

Figure S5. Flowchart building the sequence of computing steps required for implementing the activation function of a BNN neuron, i.e., the comparison with a threshold (TH), when using the 2-SIMPLY operation (i.e., 2-SIMPLY-baseline in Figure 11). The formula for the comparison operation is reported together with the scaling of the number of computing steps (i.e., #steps) as a function of the number of compared input bits (n).

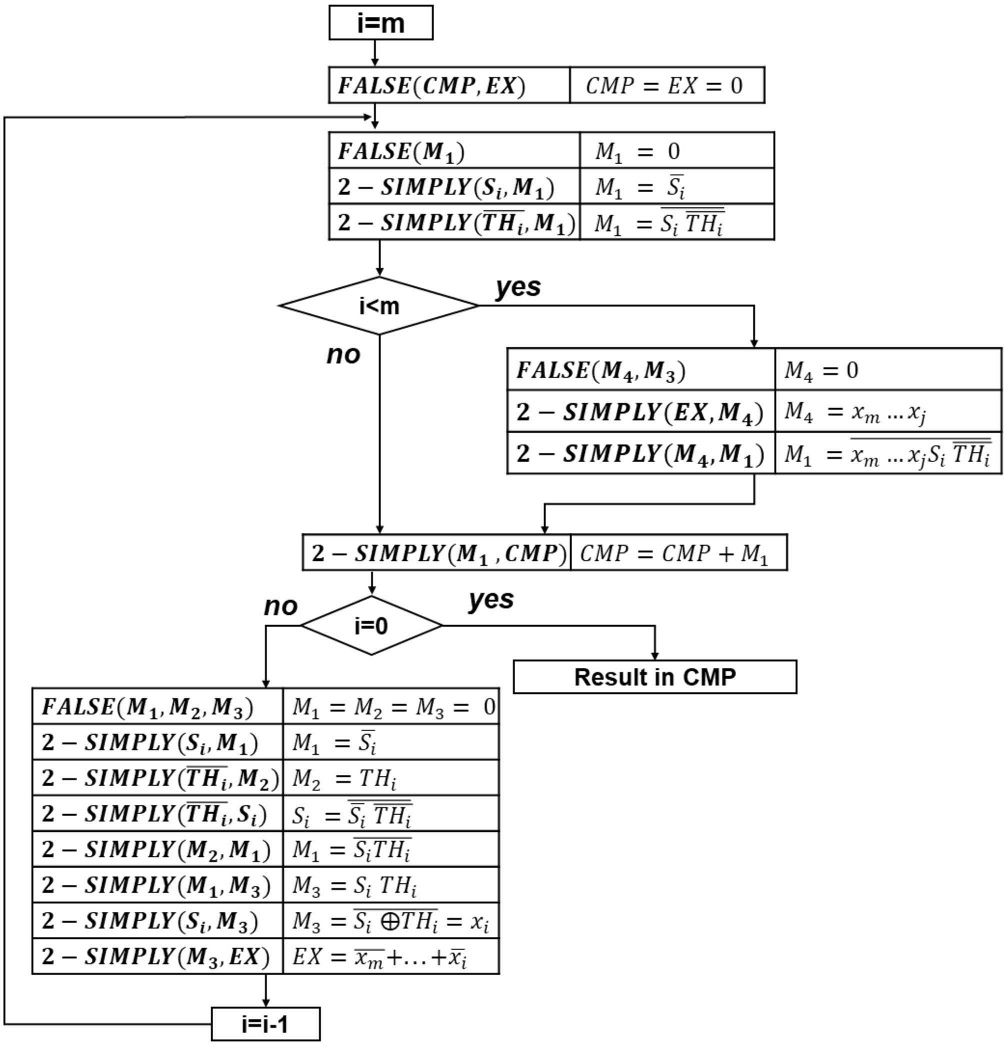

$n = m + 1$

$if\ n = 1 \rightarrow \#\boldsymbol{Steps} = \mathbf{5}$

$else\ \rightarrow \#\boldsymbol{Steps} = \mathbf{18}\boldsymbol{n} - \mathbf{12}$

Figure S6. Flowchart building the sequence of computing steps optimized for large number of input bits, that is used to implement the activation function of a BNN neuron, i.e., the comparison with a threshold (TH), when using the 2-SIMPLY operation (i.e., 2-SIMPLY Opt. Wide Words in Figure 11). The formula for the comparison operation is reported together with the scaling of the number of computing steps (i.e., #steps) as a function of the number of compared input bits (n).

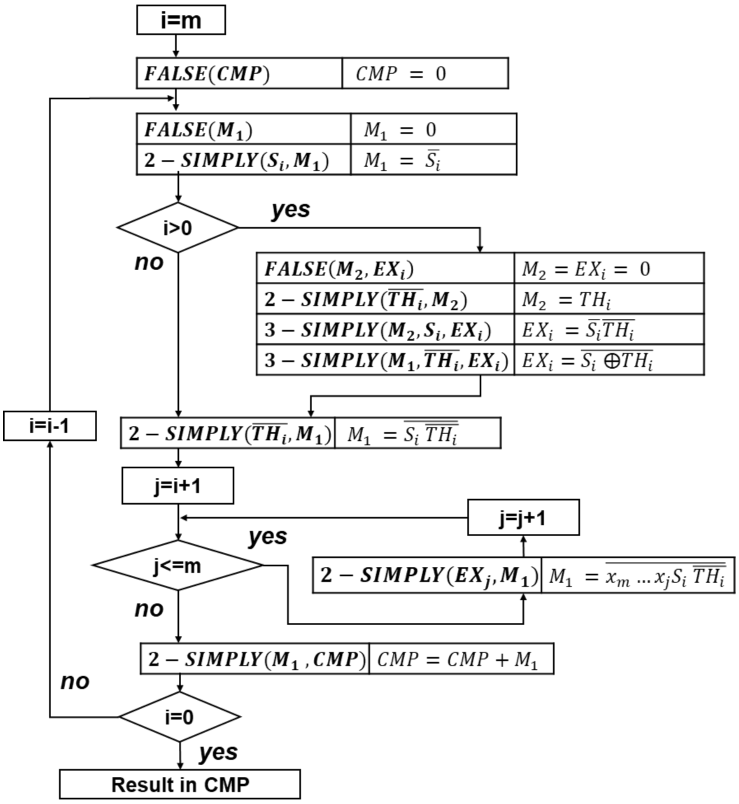

$n = m + 1$

$\#\boldsymbol{Steps} = \mathbf{0.5}\,\boldsymbol{n}^{\mathbf{2}} + \mathbf{8.5}\boldsymbol{n} - \mathbf{4}$

Figure S7. Flowchart building the sequence of computing steps that is used to implement the activation function of a BNN neuron, i.e., the comparison with a threshold (TH), when using the 3-SIMPLY operation and optimizing only the steps required for computing the intermediate XNOR operations (i.e., 3-SIMPLY Only XNOR in Figure 11). The formula for the comparison operation is reported together with the scaling of the number of computing steps (i.e., #steps) as a function of the number of compared input bits (n).

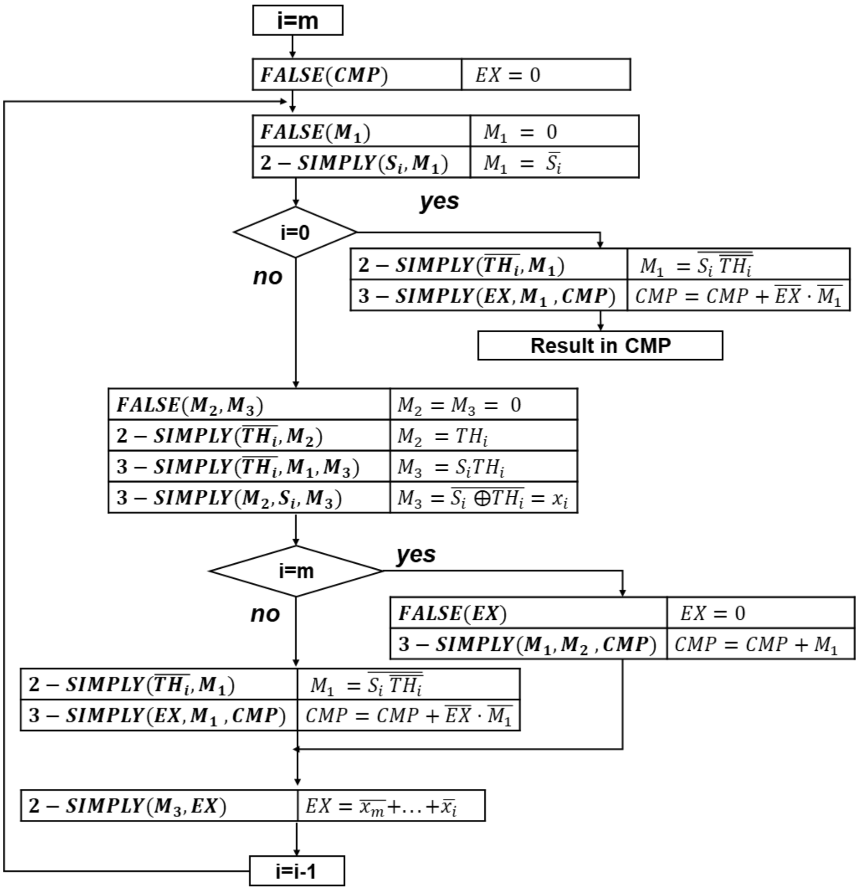

$$n = m + 1$$

$$if\ n = 1 \rightarrow \#\mathbf{Steps} = \mathbf{5}$$

$$if\ n = 2 \rightarrow \#\mathbf{Steps} = \mathbf{15}$$

$$else\ \rightarrow \#\mathbf{Steps} = \mathbf{10n - 5}$$

Figure S8. Flowchart building the optimized sequence of computing steps that is used to implement the activation function of a BNN neuron, i.e., the comparison with a threshold (TH), when using the 3-SIMPLY operation (i.e., 3-SIMPLY Opt. in Figure 11). The formula for the comparison operation is reported together with the scaling of the number of computing steps (i.e., #steps) as a function of the number of compared input bits (n).

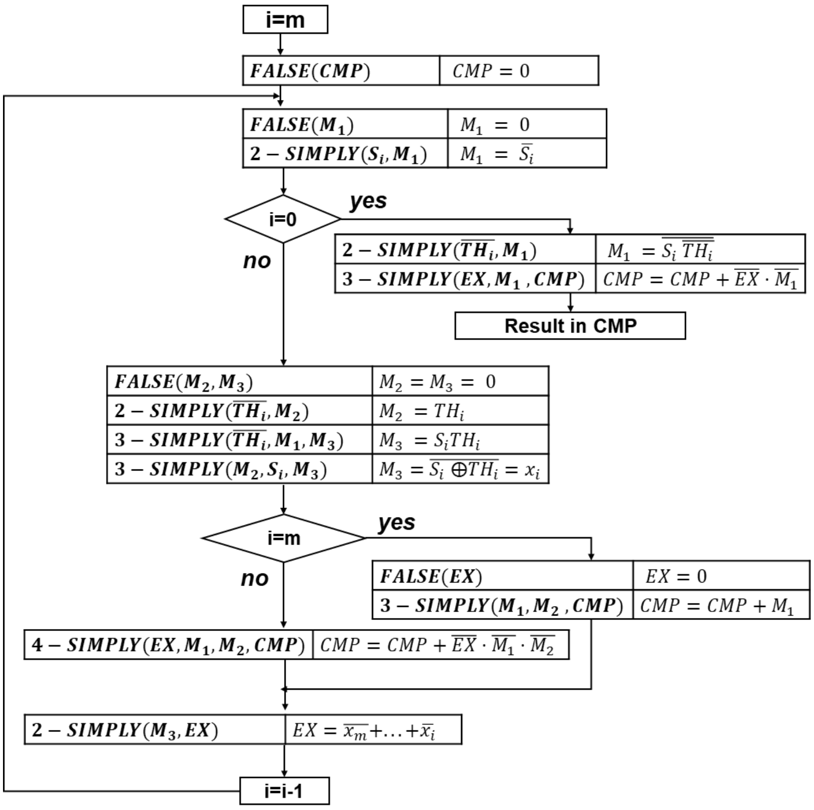

$n = m + 1$

$if\ n = 1 \rightarrow \#\boldsymbol{Steps} = \mathbf{5}$

$if\ n = 2 \rightarrow \#\boldsymbol{Steps} = \mathbf{15}$

$else\ \rightarrow \#\boldsymbol{Steps} = \mathbf{9}\boldsymbol{n} - \mathbf{3}$

Figure S9. Flowchart building the optimized sequence of computing steps that is used to implement the activation function of a BNN neuron, i.e., the comparison with a threshold (TH), when using the 4-SIMPLY operation (i.e., 4-SIMPLY Opt. in Figure 11). The formula for the comparison operation is reported together with the scaling of the number of computing steps (i.e., #steps) as a function of the number of compared input bits (n).
